# Maximizing PHB content in *Synechocystis *sp*.* PCC 6803: a new metabolic engineering strategy based on the regulator PirC

**DOI:** 10.1186/s12934-020-01491-1

**Published:** 2020-12-22

**Authors:** Moritz Koch, Jonas Bruckmoser, Jörg Scholl, Waldemar Hauf, Bernhard Rieger, Karl Forchhammer

**Affiliations:** 1 Interfaculty Institute of Microbiology and Infection Medicine Tübingen, Eberhard-Karls-Universität Tübingen, Tübingen, Germany; 2 Wacker-Chair of Macromolecular Chemistry, TUM Department of Chemistry, Technical University of Munich, Munich, Germany

**Keywords:** Cyanobacteria, PHB, Metabolic engineering, *Synechocystis* 6803, Biopolymers, Sustainable

## Abstract

**Background:**

PHB (poly-hydroxy-butyrate) represents a promising bioplastic alternative with good biodegradation properties. Furthermore, PHB can be produced in a completely carbon–neutral fashion in the natural producer cyanobacterium *Synechocystis *sp. PCC 6803. This strain has been used as model system in past attempts to boost the intracellular production of PHB above ~ 15% per cell-dry-weight (CDW).

**Results:**

We have created a new strain that lacks the regulatory protein PirC (product of *sll0944*), which exhibits a higher activity of the phosphoglycerate mutase resulting in increased PHB pools under nutrient limiting conditions. To further improve the intracellular PHB content, two genes involved in PHB metabolism, *phaA* and *phaB,* from the known producer strain *Cupriavidus necator*, were introduced under the control of the strong promotor P*psbA2*. The resulting strain, termed PPT1 (Δ*pirC*-RE*phaAB*), produced high amounts of PHB under continuous light as well under a day-night regime. When grown in nitrogen and phosphorus depleted medium, the cells produced up to 63% per CDW. Upon the addition of acetate, the content was further increased to 81% per CDW. The produced polymer consists of pure PHB, which is highly isotactic.

**Conclusion:**

The amounts of PHB achieved with PPT1 are the highest ever reported in any known cyanobacterium and demonstrate the potential of cyanobacteria for a sustainable, industrial production of PHB.

## Introduction

The global contamination with non-degradable plastic is a huge environmental burden of our time [15, 29]. While bioplastics have been suggested as potential solution, they still represent only a very small fraction of the plastics overall used [11]. Furthermore, many of these bioplastics have unsatisfying biodegradation properties. The most common bioplastic, PLA (poly-lactic-acid), is barely degraded in marine environments [34]. This has led to an increasing interest into another class of bioplastics with improved degradation properties: poly-hydroxy-alkanoates (PHAs). The most common variant of this chemical class is poly-hydroxy-butyrate (PHB) which is produced by various microorganisms. Currently, PHB is produced by fermentation using heterotrophic bacteria, such as *Cupriavidus necator* or *Escherichia coli *[6]. However, these production processes require crop-derived organic carbon sources for growth and production and pose a threat to human food-supply. An alternative strategy to produce PHB independently of cropland use, is the usage of phototrophic organisms, such as cyanobacteria [1, 3]. *Synechocystis *sp*.* PCC 6803 (hereafter *Synechocystis*) is a well-studied model organism for phototrophic growth and a natural producer of PHB [14, 45]. Under conditions of nutrient limitation, for example nitrogen starvation, the cells enter into a resting state in a process that is known as chlorosis [2]. During chlorosis, cyanobacteria do not only degrade their photosynthetic apparatus, but also accumulate large quantities of glycogen as a carbon- and energy-storage [8, 23]. During the late stages of chlorosis, the cells start to degrade glycogen and convert it to PHB [26]. However, the intracellular amount of PHB in chlorotic cells remains rather low and only represents about 10–20% of the cell dry weight (CDW). A recent economic analysis suggests that one of the factors that make the production of PHB in cyanobacteria less attractive than that in heterotrophic organisms is the low ratio of PHB/CDW in cyanobacteria [24]. One major goal is therefore, to optimize cyanobacteria so that they accumulate higher intracellular levels of PHB. This would not only increase the yield but also simplify the downstream-process of extracting PHB from the cells.

In the past, there have been various attempts to further boost the amount of PHB in cyanobacterial cells. A selection of the most important approaches is listed in Table 1.

**Table 1:** Previous attempts to optimize the medium or genetic background of *Synechocystis sp*. PCC 6803 for the production of PHB. Further approaches (also in other cyanobacteria) have been reviewed recently [19]

Genotype	PHB content (% CW)	substrate	Production condition	Polymer composition	References
WT	29	0.4% acetate	–P	PHB	[38]
overexpression *phaAB* (native)	35	0.4% acetate	–N	PHB	[21, 22]
overexpression *phaABC* (*Cupriavidus necator*)	11	10 mM acetate	–N	PHB	[42]
overexpression nphT7, phaB, phaC	41	0.4% acetate	Limited air exchange, –N	–	[28]
overexpression Xfpk	12	CO_2_	–N, –P	PHB	[5]
overexpression *sigE*	1.4	CO_2_	–N	PHB	[36]
overexpression *rre37*	1.2	CO_2_	–N	PHB	[37]

Most of them have focused on genetic engineering strategies to reroute the intracellular flux towards PHB [5, 28, 36, 37]. *Synechocystis* naturally produces PHB from acetyl-CoA via the enzymes acetyl-CoA acetyltransferase (PhaA), acetoacetyl-CoA reductase (PhaB) and the heterodimeric PHB synthase (PhaEC). The overexpression of the genes encoding for these enzymes is known to increase the PHB content within the cells [21, 42].

The highest rate of photosynthetically produced PHB in a wild type (WT) cyanobacterium was reported for a strain isolated from a wet volcanic rock in Japan. In this strain, *Synechococcus *sp*.* MA19, PHB constituted 27% of the CDW [32]. It has to be mentioned, though, that no other group was ever able to obtain this strain from a laboratory or a strain collection repository [31]. Another valuable approach turned out to be random mutagenesis via UV radiation [18]. This yielded a strain, *Synechocystis sp*. PCC 6714, that produced PHB up to 37% of the CDW under phototrophic growth with CO_2_ as the sole carbon source.

Besides genetic engineering approaches, optimization of growth and medium conditions was also demonstrated to increase PHB production [38]. A study investigating 137 different cyanobacterial species found that 88 of them produced PHB when the growth medium was deprived of a specific nutrient [16]. The highest yields were often achieved when cells were starved for nitrogen [16], but also the addition of organic carbon sources, like acetate or fructose, resulted in increased PHB production [38]. Conflicting results were reported from attempts to increase PHB synthesis in cells grown under conditions of limited gas exchange. Whereas some groups reported increased yields [28, 38], other groups failed to reproduce this effect [20]. In agreement with that, a recent study demonstrated that cells grown under static conditions and, thereby, exposed to limited gas-exchange, exhibited a decreased PHB accumulation [25]. A comprehensive overview about these strategies can be found in recent reviews [19, 41]. Despite the various approaches to further increase the PHB content in *Synechocystis*, the highest PHB levels reached so far are still far below those obtained in heterotrophic bacteria, in which more than 80% of biomass is converted into the desired product.

We have recently identified in *Synechocystis* a gene, *sll0944*, which plays a key role in the partitioning of newly fixed CO_2_. It encodes a small protein, termed PirC (PII-interacting regulator of **c**arbon metabolism) that acts as inhibitor of the 2,3-phophoglycerate-independent phosphoglycerate mutase (PGAM) and whose action on PGAM is controlled by the signal processor protein PII. Under nitrogen-sufficient conditions, corresponding to low 2-oxoglutarate levels, PirC is sequestered in a complex with PII. However, under nitrogen-limiting conditions, corresponding to elevated 2-oxoglutarate levels, PII releases PirC, causing inhibition of PGAM activity. Consequently, the metabolic flux towards lower glycolysis is tuned down and most newly fixed carbon is converted to glycogen. The effect that *sll00944* triggers glycogen accumulation was independently described by another group [33]. Further, we observed that the PirC-deficient mutant over-accumulated PHB during nitrogen-starvation, which agrees with a higher PGM activity due to lacking PirC inhibition [35]. These findings identified PirC as a toggle switch for the direction of carbon flow into lower glycolysis, from where acetyl-CoA and related metabolites are derived and suggested useful applications in metabolic engineering of cyanobacteria. Therefore, the aim of this study was to maximize PHB content by combining the deletion of *pirC* with other factors known to improve PHB synthesis. This resulted in a strain that can accumulate more than 80% of PHB and is by far the most efficient PHB producing oxygenic photosynthetic organism reported to date.

## Results

**Fig. 1:**
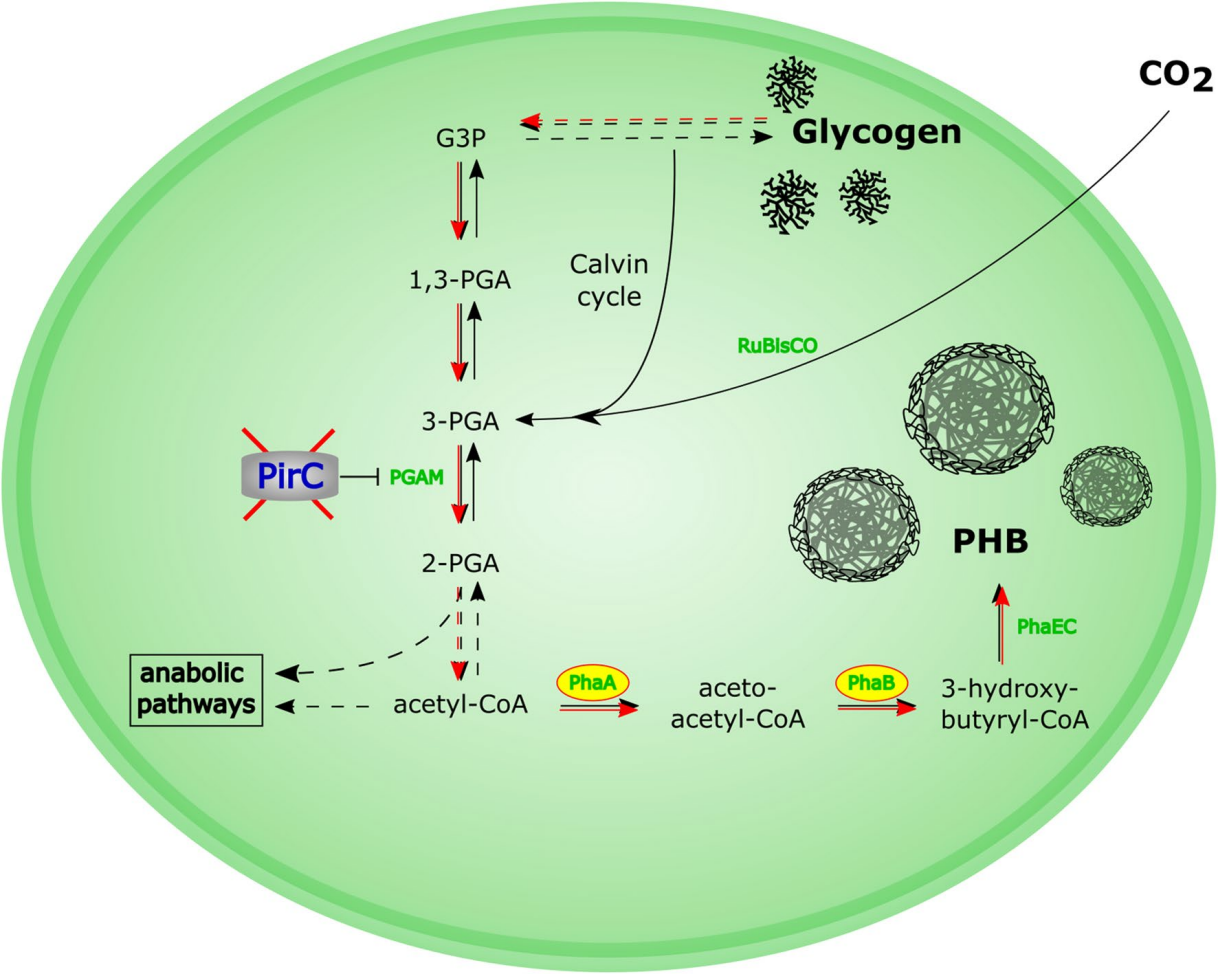
Overview about important carbon-pathways in *Synechocystis* and alterations of PPT1 compared to the WT. PPT1 contains a deletion of p*irC*, as well as an overexpression of *phaA* and *phaB* from *C. necator*. Important enzymes are shown in green. Increased fluxes in PPT1 compared to the WT are indicated by red arrows

Recently, it was shown that overexpression of the PHB synthase PhaEC in *Synechocystis* PCC 6803 led to a reduction in PHB production, while overexpression of the endogenous *phaAB* genes caused an increase in intracellular accumulation of PHB [21]. To test if the PHB content of a *Synechocystis *sp*.* PCC 6803 *pirC* mutant strain (Δ*pirC*) could be further increased, we cloned and overexpressed *phaA* and *phaB* from the PHB producer strain *Cupriavidus necator* (formerly known as *Ralstonia eutropha*) into Δ*pirC*. We used these genes, since *C. necator* is known as a highly efficient PHB synthesizing organism. Furthermore, the expression of heterologous enzymes ensures that they are not inhibited by intracellular post-transcriptional regulatory mechanisms. Both genes were cloned into a pVZ322 vector under the control of a strong promotor, P*psbA2*. The plasmid was then transformed into the strain Δ*pirC*, thereby creating the strain Δ*pirC*-RE*phaAB* (Fig. 1). For the sake of clarity, the strain is referred to as PPT1 (for *PHB Producer Tübingen 1*) from here on.

### Strain characterization

**Fig. 2:**
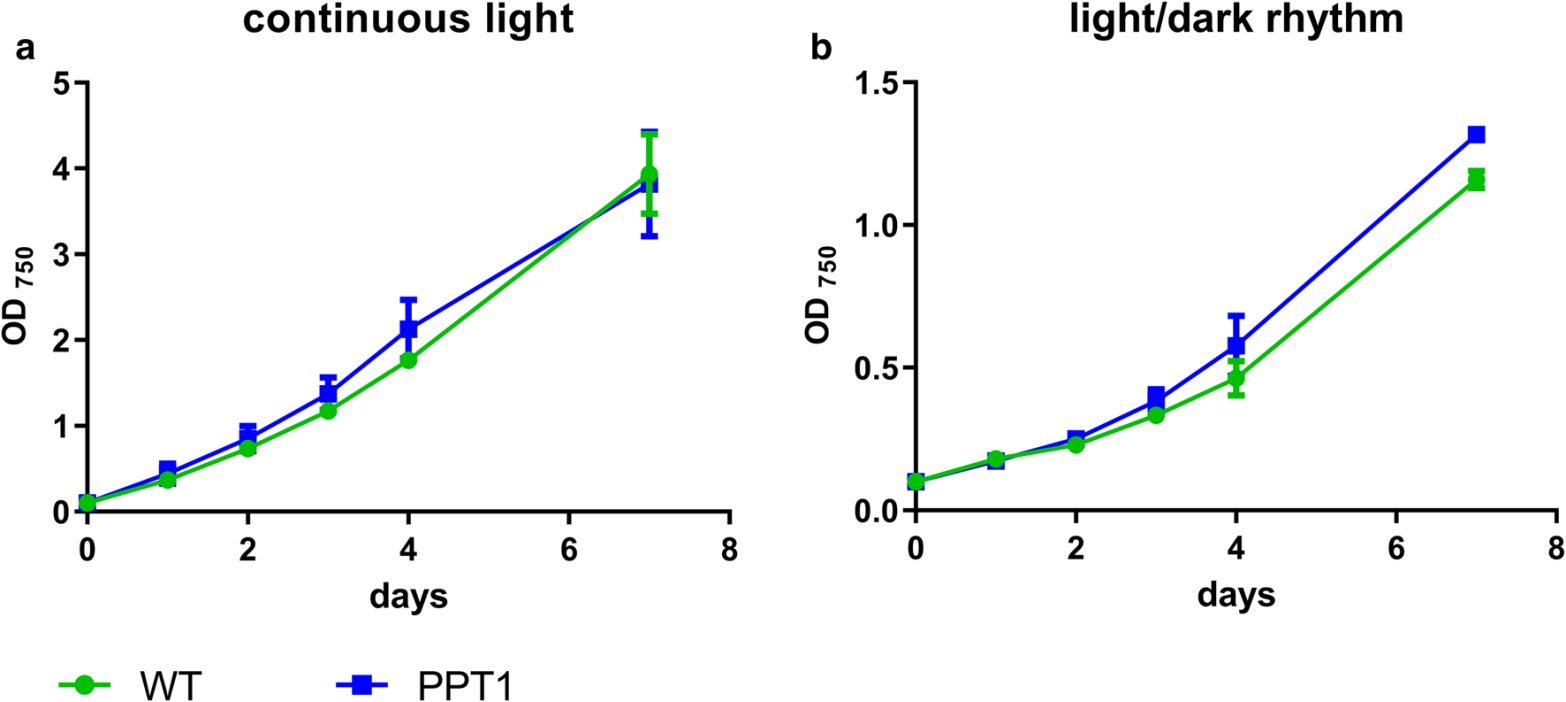
Growth behavior of WT and PPT1 strains under continuous illumination (**a**) or under a 12/12 h light/dark regime (**b**). Growth was determined over 7 days by recording the OD_750_. Each point represents a mean of three independent biological replicates

To compare the growth of the newly generated strain with the WT, both strains were grown under continuous illumination as well as under a 12/12 h light/dark regime (Fig. 2).

Under both light regimes, WT and PPT1 strain exhibited similar growth rates. This was also the case when the strains were grown on solid agar plates (Additional file 1: Figure S1). To test whether the mutant strain was able to produce PHB during vegetative growth, we assessed PHB production in both strains during exponential and stationary growth phase on BG_11_ medium (OD_750_  ~ 1 and ~ 3, respectively) (Additional file 2: Figure S2). While the WT did not produce any detectable amount of PHB during exponential growth, in the mutant PHB constituted ~ 0.5% of the CDW. Under stationary conditions, none of the strains produced any detectable amount of PHB.

**Fig. 3:**
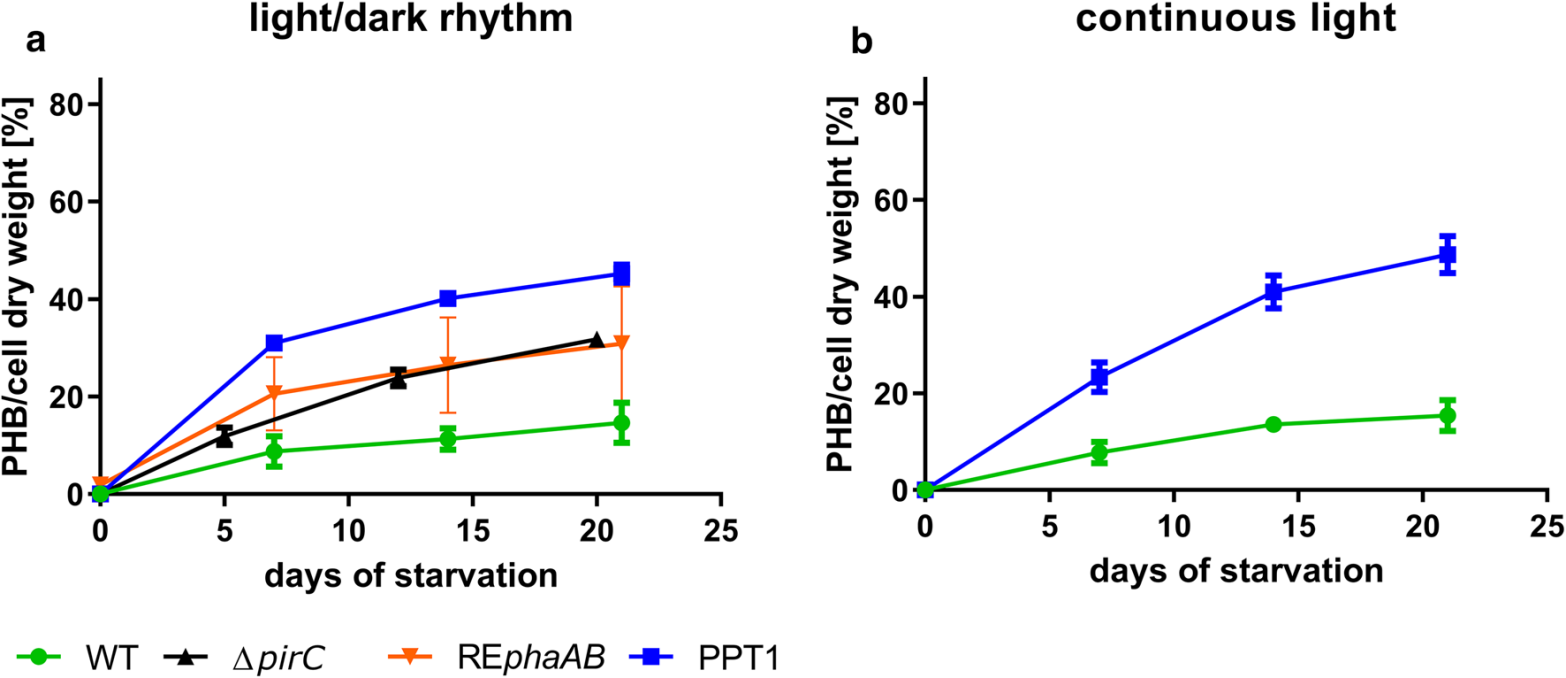
PHB content of WT (blue), Δ*pirC* (black), RE*phaAB* (orange) and PPT1 (green) cells cultivated under different light regimes. Exponentially grown cells were shifted to nitrogen free BG_0_ and cultivated under either diurnal (12 h light/12 h darkness) (**a**) or continuous light (**b**). Each point represents a mean of three independent biological replicates

To test, whether the newly generated mutant was able to accumulate higher amounts of PHB during nitrogen starvation, i.e. chlorosis, different growth conditions were systematically tested. First, the impact of continuous illumination compared to day-night cycles was tested. To this aim, WT and PPT1 cells were shifted to nitrogen-free BG_0_ medium to induce chlorosis and were subsequently grown under 12/12 h light /dark cycle or under continuous illumination. The intracellular amount of PHB was quantified and normalized to the CDW in all conditions (Fig. 3). For an easier comparison, all following graphs about PHB accumulation have the same y-axis scale.

To test the influence of the individual genetic modifications, the PHB content of two strains harbouring only one of the two genetic alterations (Δ*pirC* or RE*phaAB*, respectively) was measured. Compared to the WT, both the Δ*pirC* and the RE*phaAB* strains produced higher amounts of PHB (32 and 31% per CDW, respectively) after three weeks of chlorosis. When both genetic changes were combined (PPT1), the accumulation of PHB was further increased to 48 and 45% PHB/CDW in dark/light or continuous light, respectively. With 31% of PHB per CDW after 7 days in diurnal cultivation, the initial rate of PHB synthesis in the PPT1 cells was higher as compared to continuous illumination, in which conditions PHB amounted to 23% of the CDW. Therefore, these conditions were used for further experiments.

### Medium optimization

**Fig. 4:**
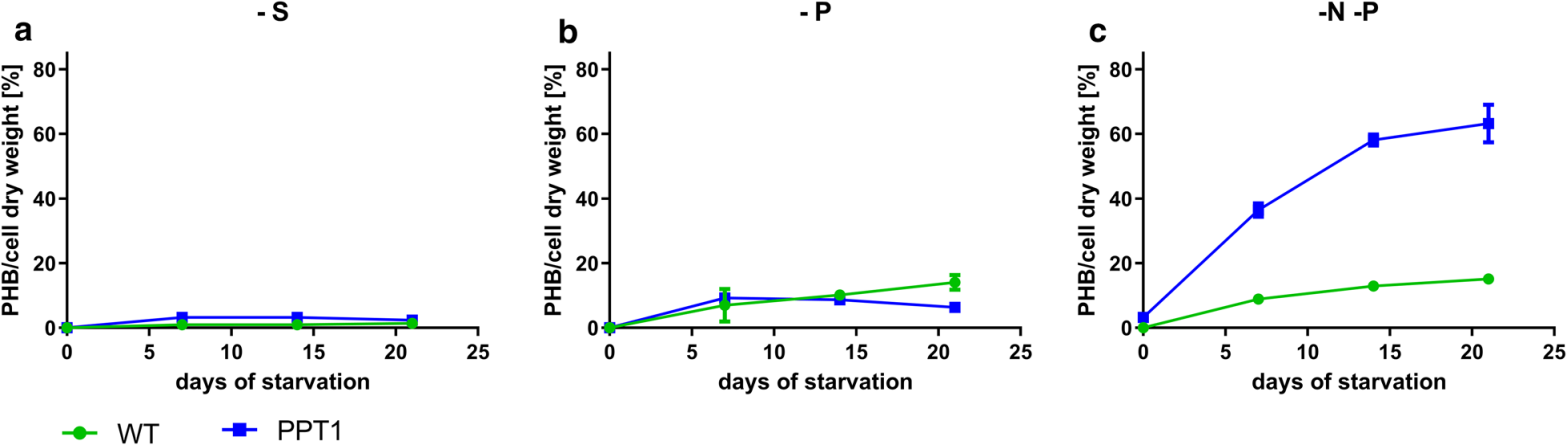
PHB content of WT (green) and PPT1 (blue) cells grown in different media under dark/light rhythm. To induce PHB production, exponentially grown cells were shifted to either sulphur, phosphorus or nitrogen/phosphorus-free medium (**a**, **b** and **c**, respectively). Each point represents a mean of three independent biological replicates

Other studies have reported that, besides nitrogen, the lack of other elements can induce the biosynthesis of PHB in *Synechocystis* [16]. To test this effect on the newly generated strain, WT and PPT1 cells were shifted to either sulphur, phosphorus or nitrogen/phosphorus-free medium and the intracellular content of PHB was quantified (Fig. 4).

Whenever phosphate free conditions were used, the pre-cultures of *Synechocystis* cells were already grown in phosphate-free BG_11_, in order to deplete the intracellular storage pools of polyphosphate. In sulphur- as well as in phosphorus-free medium, both the WT and the PPT1 strain produced only minor amounts of PHB. However, when the cells were shifted to nitrogen/phosphorus-free medium for three weeks, the mutant strain accumulated higher amounts of PHB than the WT (up to 63% and 15% of the CDW, respectively). All further experiments were, hence performed, in nitrogen- and phosphorus depleted BG_11_ medium.

**Fig. 5:**
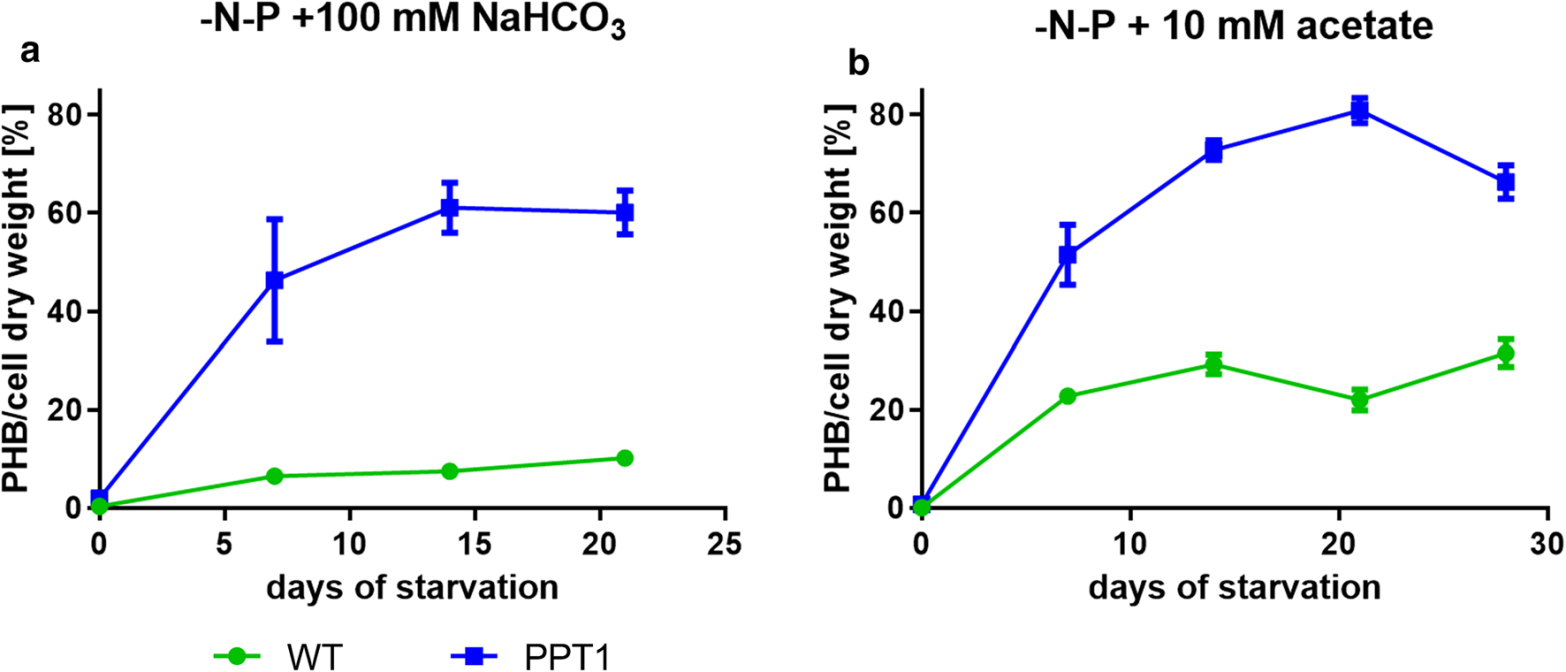
PHB production of WT (green) and PPT1 (blue) cells grown under alternating light/dark regime. **a** Cells shifted to nitrogen/phosphorus free medium with the addition of 100 mM NaHCO_3_. **b** Cells shifted to nitrogen/phosphorus free medium with the addition of 10 mM acetate. Each point represents a mean of three independent biological replicates

To test if the PHB levels could be further increased by the addition of an additional carbon source, either 100 mM NaHCO_3_ or 10 mM acetate was added after the shift to nitrogen/phosphorus-free medium (Fig. 5).

As in the previous experiments, cells were again cultivated in diurnal light/dark regime. When NaHCO_3_ was added, the PPT1 cells reached an intracellular PHB content of up to 61% per CDW after two weeks, while the WT accumulated only 10% of PHB per CDW. Notably, the initial production rate was further increased in the PPT1, leading to an average 46% of PHB per CDW already after one week. When, instead of NaHCO_3_, 10 mM acetate was added, the WT reached an intracellular PHB content of up to 32% per CDW after four weeks, while the PPT1 mutant accumulated up to 81% per CDW after three weeks of starvation (Fig. 5a). An additional week of starvation did not further increase the yield, but instead slightly reduced it. When cells were grown under the same conditions but with continuous illumination, the amounts of PHB were much lower (Additional file 3: Figure S3).

To test if the limitation of gas exchange could lead to a further increase in PHB production, nitrogen-phosphorus starved cells were grown in sealed vessels. Despite an initial increase in the intracellular levels of PHB, these strongly dropped and remained low until the end of the cultivation period (Additional file 4: Figure S4).

### Visualization of PHB granules

**Fig. 6:**
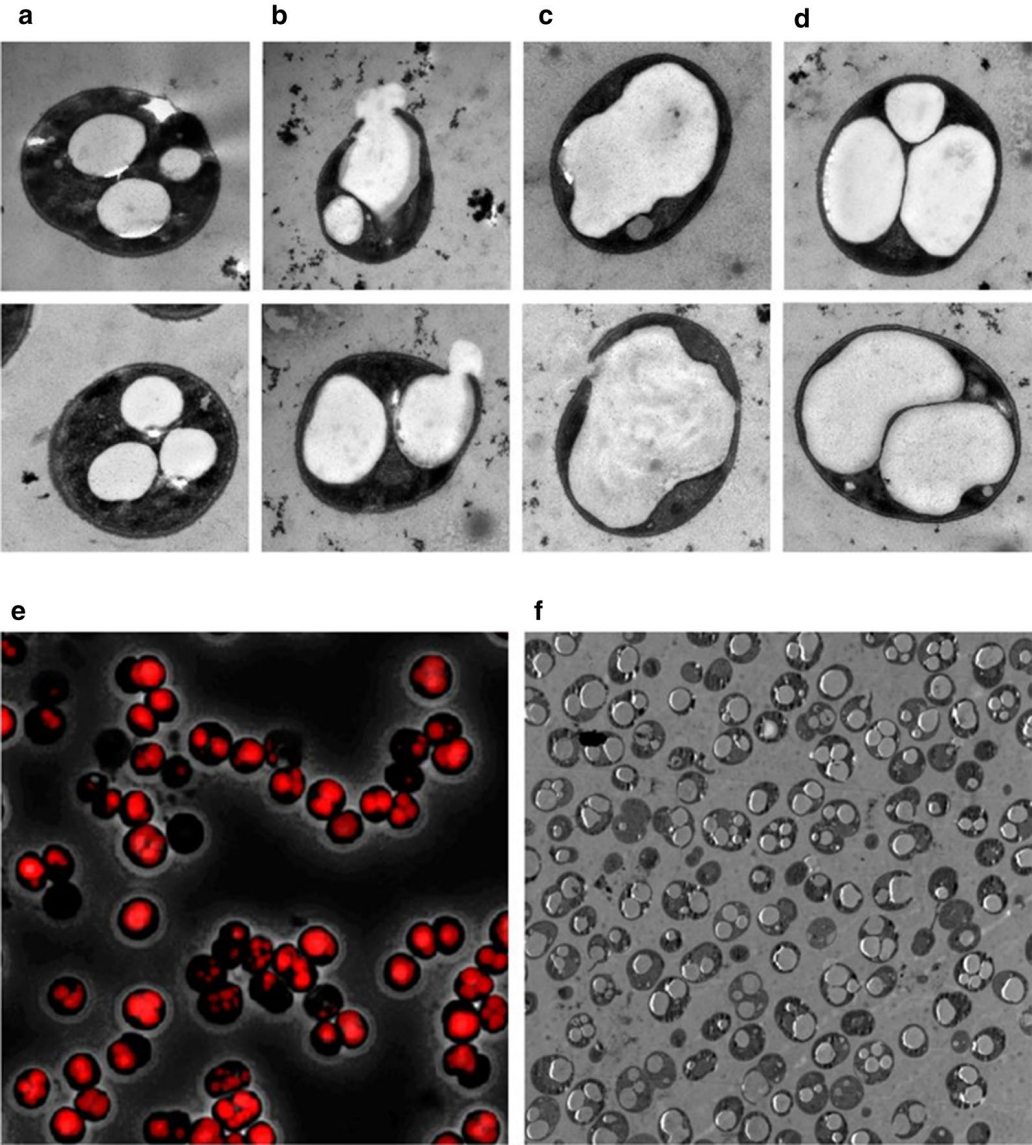
WT (**a**) and PPT1 cells (**b**–**f**) after 21 days of nitrogen-phosphorus-starvation with 10 mM acetate grown under alternating light/dark regime. **a** WT cells for comparison. **b** PTT1 cells showing a ruptured cell wall. **c** PPT1 cells with a single PHB granule. **d** PPT1 cells with multiple granules. **e** Fluorescence microscopic picture of PPT1 cells; PHB granules are visualized as red inclusions after staining with Nile red. **f** Overview of multiple PPT1 cells

To find out whether the increased intracellular levels of PHB affected the morphology of the cells, and how these masses of PHB organized within the cells, we performed fluorescence as well as transmission-electron microscopy (TEM) analysis (Fig. 6) on the same PPT1 cells that were used for PHB quantification in Fig. 5b (21 days of growth under nitrogen-phosphorus starvation with 10 mM acetate). The electron-microscopic images indicated that the cells were fully packed with PHB granules, although some heterogeneity among cells was visible (Fig. 6c, d). Interestingly, most cells contained not multiple, but only one large PHB granule, suggesting fusion of smaller granules into a large one. In several cases, the cells were ruptured and released PHB into the environment (Fig. 6b). From the overview of the TEM pictures shown in Fig. 6f, it becomes apparent that most cells contained large quantities of PHB. The fluorescence microscopy analysis confirmed the results obtained with TEM (Fig. 6e).

### Qualitative analysis of PHB

**Fig. 7:**
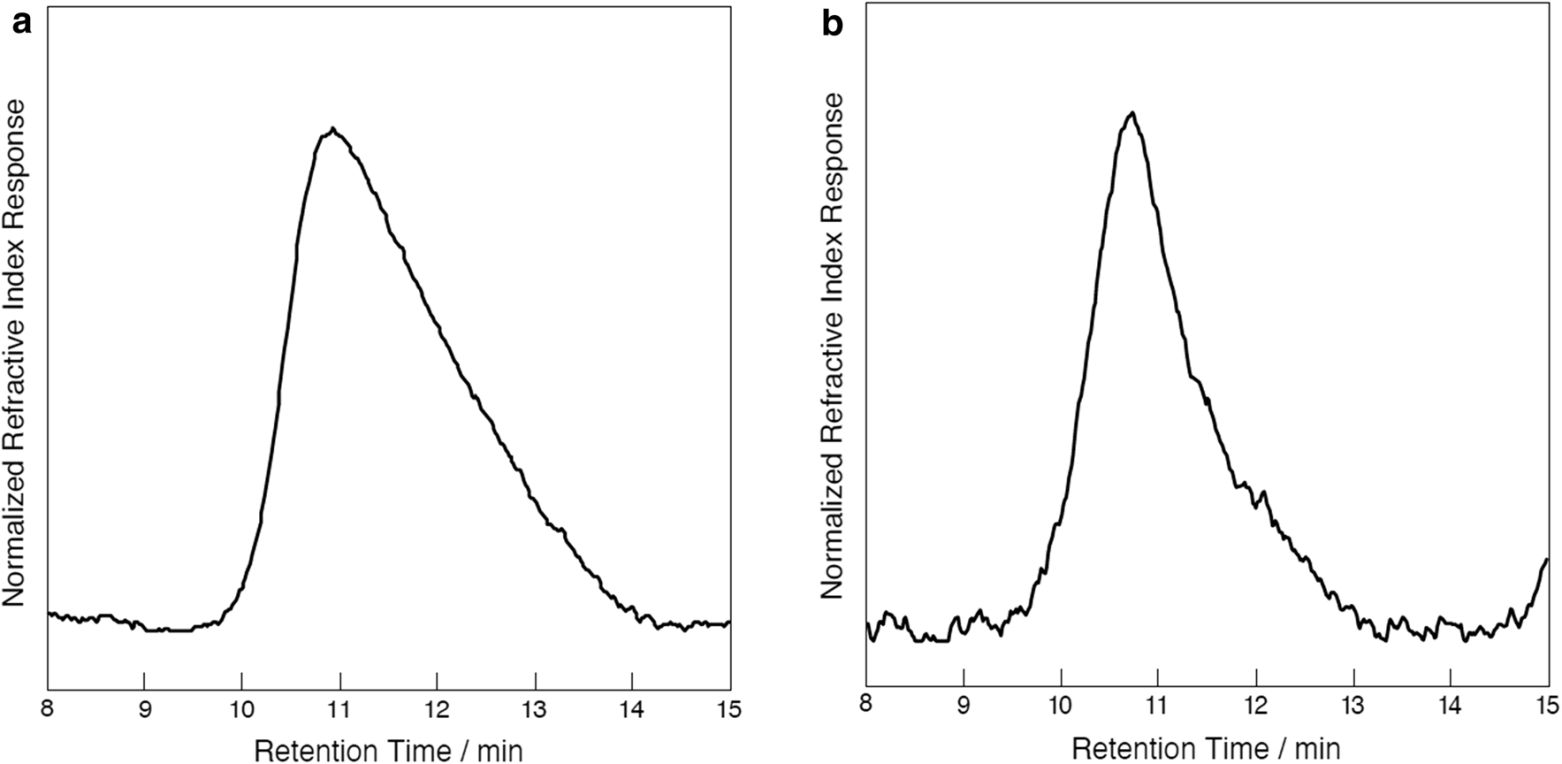
GPC analysis of PHB from an industrial standard (**a**) and PPT1 (**b**) strain

To further characterize the physico-chemical properties of the PHB produced by PPT1, these cells were cultivated for four weeks under nitrogen and phosphorus starvation. They were then broken with sodium hypochlorite and the purified PHB was analysed via gel permeation chromatography (GPC), 1H- and 13C-Nuclear Magnetic Resonance (NMR), to determine the molecular weight, the dispersity, the purity and the tacticity of the polymer, respectively. GPC analysis revealed that PPT1 produces a high-molecular-weight polymer with relatively narrow dispersity and average molecular weight of Mn = 503 kg/mol (*Ð* = 1.74), which was more than twice as high than the control (Mn = 246 kg/mol, *Ð* = 2.33) (Fig. 7).

1H and 13C NMR spectroscopy analysis confirmed that the polymer consisted of completely pure PHB (Additional file 5: Figure S5, Additional file 6: Figure S6). Furthermore, the observed singlet resonances in the 13C NMR spectrum indicated that the PHB derived from PPT1 is highly isotactic (Additional file 7: Figure S7).

## Discussion

### ΔpirC-REphaAB produces maximum amounts of PHB

As previous studies have shown, PHB is derived from the intracellular glycogen pools [26]. Furthermore, the carbon flux is regulated by the protein PirC through controlling the activity of the PGAM reaction, which convert 3-PGA to 2-PGA and thereby, directs carbon-flow into lower glycolysis [35]. Deletion of *pirC* results in a strong increase of glycogen catabolism during prolonged nitrogen starvation. The simultaneous expression of the genes *phaA* and *phaB,* redirects most of acetyl-CoA pool towards the synthesis of PHB. Since PhaB catalyzes the conversion of one NADPH to NADP, the reaction yielding hydroxybutyryl-CoA is strongly favored during nitrogen starvation, when the pool of NADPH is increased [13], pushing the synthesis of PHB forward.

When grown in nutrient-replete balanced medium, the growth behavior of the PPT1 strain was comparable to that of the WT, in liquid medium as well as on solid agar plates (Fig. 2, Additional file 1: Figure S1). This was expected since there was hardly any PHB produced during vegetative growth (Additional file 2: Figure S2) due to the consumption of the metabolites derived from lower glycolysis in anabolic reactions for cell growth. The simultaneous deletion of *pirC* and overexpression of the *C. necator phaAB* genes in PPT1 acted additively with respect to PHB production under nitrogen starvation, as PHB levels in this strain reached levels well above those of the individual strains, i.e. Δ*pirC* and RE*phaAB* (Fig. 3a). Similar intracellular amounts of PHB were reached regardless of the light regime, indicating that the production of PHB is not limited by the availability of light in PPT1 (Fig. 3). The accumulation of PHB was further boosted by combined nitrogen-phosphorus starvation (Fig. 4c). This is in accordance with previous studies, reporting that combined nitrogen-phosphorus starvation leads to the highest PHB production [5]. In contrast, the individual depletion of either sulfur or phosphorus resulted only in a small intracellular accumulation of PHB (Fig. 4a, b). It was shown before, that nitrogen limitation is the most efficient condition for the induction of PHB synthesis in cyanobacteria [16]. In a recently created strain though, it was shown that a random mutation in a phosphate specific membrane protein PstA causes a strong increase in PHB accumulation, hinting towards the importance of phosphorus for PHB production [17].

When 100 mM NaHCO_3_^−^ was added to PPT1 cells cultivated in nitrogen-phosphorus depleted medium, a further increase in intracellular PHB levels was reached in the initial production phase. This indicates that a limitation of carbon was impairing further PHB production in the previous conditions. The addition of high amounts of NaHCO_3_^−^ is beneficial for the remaining CO_2_-fixation, thereby replenishing the metabolite pools that are consumed by PHB synthesis [9]. Notably, one of the three biological replicates exhibited a PHB content of 61%/CDW after one week, indicating the potential to accelerate the pace of PHB formation via carbon supplementation. The overall PHB content was further increased by the addition of 10 mM acetate, hinting towards a limitation of the precursor acetyl-CoA. Since acetate can be converted to acetyl-CoA in a single enzymatic reaction, it is more efficiently metabolized to PHB than NaHCO_3_.

Interestingly, the highest PHB content was reached under light/dark regime, while its accumulation was strongly diminished under continuous light, even upon the addition of acetate (Fig. 5 and Additional file 3: Figure S3). This is in agreement with previous observations showing that cultivation under diurnal light/dark cycles increased PHB production [25]. Cells which were cultivated under conditions of gas exchange limitation displayed reduced PHB accumulation. This was also reported by other groups [20] and might be due to the lack of oxygen during the night, which is necessary for maintaining cell metabolism. Alternatively, excess of oxygen during the day could increase the oxygenase reaction with a consequent waste of energy and decline in cell metabolism.

### Morphology of PHB granules

TEM pictures showed *Synechocystis* PPT1 cells fully packed with PHB granules (Fig. 6). Additionally, a certain number of cells displayed fractured cell envelopes, leading to extracellular leakage of PHB granules. The rupture of the cells could be due to intracellular mechanical pressure from the expanding PHB granules or it could be the result of mechanical stress during the preparation of the cells for TEM analysis. Whatever the cause, the presence of the ruptures indicates an increased cell fragility due to the massive accumulation of PHB, since this effect was not detected in WT cells, which contained less PHB but were processed in the same way as the PPT1 strain. This also indicates that some of the PPT1 cells can no longer accumulate PHB without severely compromising cell viability. It was previously hypothesized that *Synechocystis* cells cannot accumulate large quantities of PHB due to steric hindrance of the thylakoid membranes. This study demonstrates the opposite. Interestingly, the majority of the cells that contained large PHB-quantities only possessed very few granules, often just one single granule. This indicates that PHB granules merge together once they exceed a certain size.

### Qualitative analysis of PHB

While other bacteria are able to produce PHAs with mixed side chains, such as 3-hydroxyvalerate, analysis of the polymer extracted from PPT1 cells revealed that it consists of PHB only. This indicates that the *Synechocystis* PhaEC enzyme selectively produces PHB. In the future, a mutant strain harbouring a heterologous PHA polymerase could be generated for the production of heteropolymers with improved material properties, such as poly(3-hydroxybutyrate-*co*-3-hydroxyvalerate) (PHBV). Such a PHA polymerase has been shown to be present in other cyanobacteria, like *Nostoc microscopicum* [43]. In previous analyses the average molecular weight of PHB from *Synechocystis* and *Synechocystis* sp. PCC 6714 was determined at Mn ~ 130 and 316 kg mol^−1^, respectively [27, 37]. Compared to these, the PHB derived from PPT1 with an average weight of 503 kg/mol is high-molecular. It is also highly isotactic, which suggests it is well biodegraded.

### Conclusiosn and outlook

To further accelerate PHB production, overexpressing a strongly processive PHB-polymerase could be beneficial. Although it was shown that higher levels of PhaEC can lower the PHB content [22], its activity could become rate limiting once PHB levels as high as those obtained in the present study are reached. The insertion of another short-chain-length PHA-polymerase could furthermore lead to the production of PHAs with improved material properties (PHBV). In order to improve the overall production yields, increased growth rates before depleting nitrogen and phosphorus will be necessary, for example by using high-density cultivators. In similar approaches, *Synechocystis* cultures reached OD_750_ above 50 when high light and CO_2_ concentrations were applied [7, 30]. Under those ideal conditions, up to 8 g of dry biomass l^−1^ d^−1^ were reached. If the time for chlorosis is assumed to be similar to the time required for cultivation and an intracellular PHB content of 60% is reached, 2,4 g PHB l^−1^ days^−1^ could be produced under completely phototrophic conditions. Since PHB production in the strain PPT1 is optimal under light/dark regime, the strain is also well suited for outdoor cultivation. Scaling up the cultivation to larger reactors would further reduce the production costs of PHB [39]. Additionally, the ability of autotrophic cyanobacteria to sequester CO_2_ from the atmosphere could be beneficial for CO_2_ emission trading. Alternatively, a growth-coupled PHB production could be beneficial in certain cultivation settings.

In summary, this study shows for the first time that cyanobacteria have the potential to accumulate large quantities of PHB, previously thought to be reserved to heterotrophic bacteria. Furthermore, we demonstrate that also under cultivation with CO_2_ as the sole carbon source, *Synechocystis* is able to accumulate high quantities of PHB. This is of high relevance for the sustainable production of PHB as bioplastic and lays the foundation for the industrial production of carbon neutral plastic alternatives.

## Materials and methods

### Cyanobacterial cultivation conditions

If not stated differently, *Synechocystis *sp*.* PCC 6803 cultures were grown in standard BG_11_ medium with the addition of 5 mM NaHCO_3_ [40]. The cultures were constantly shaken at 125 rpm, 28 °C and at an illumination of ~ 50  µE. A 100 ml Erlenmeyer flask was used to grow 50 ml of bacterial culture. When cells were grown under alternating light/dark rhythm (12 h each), the pre-cultures were adapted to these conditions by cultivating them under light/dark rhythm for two days. Whenever required, appropriate antibiotics were added to the mutant strains. When cultivation in depletion-medium was required, the following was used: for nitrogen starvation BG_0_ (BG_11_ without NaNO_3_; for sulfur starvation BG_11_ supplemented with MgCl instead of MgSO_4_; for phosphorus starvation BG_11_ supplemented with KCl instead of K_2_HPO_4_. Since *Synechocystis* has intracellular polyphosphate storage polymers, a preculture in phosphorus free medium was inoculated two days before the actual shift to phosphorus-free medium. For all starvations, exponentially grown cells (OD_750_ 0.4–0.8) were washed twice in the appropriate medium. For this, the cells were harvested at 4000*g* for 10 min, the supernatant discarded and the pellet resuspended in the appropriate medium. Afterwards the culture was adjusted to an OD_750_ of 0.4. For growth on solid surfaces, cells at an OD_750_ = 1 were dropped on BG_11_ plates containing 1.5% agar. A serial dilution of the initial culture was prepared in order to count individual colony-forming-units. A list of the strains used in this study is provided in Table 2.

**Table 2:** List of the strains used in this study

Name	Genotype	References
WT	*Synechcoystis *sp*.* PCC 6803	Pasteur culture collection
Δ*sll0944*	KanR	[35]
RE*phaAB*	pVZ322 with psbA2 regulated *phaAB* genes from *Cupriavidus necator*; GenR	This study
Δ*sll0944*-RE*phaAB*	KanR, GenR	This study

### Construction of RE*phaAB* and Δ*pirC*-RE*phaAB* mutants

The promoter *psbA2* and the *phaAB* genes were amplified from genomic DNA of *Synechocystis* and C*upriavidus necator*, respectively. For this, the primer psbaA2fw2/psbA2rv2 or RephaABA2fw/RephaABA2rv were used (Table 3). A Q5 high-fidelity polymerase (NEB) was used to amplify the DNA fragments. The latter were subsequently assembled into the pVZ322 vector [12], which was beforehand linearized with XbaI. The resulting vector was propagated in *E. coli* Top10 and isolated using a NEB miniprep kit. The plasmid was subsequently sequenced to verify sequence integrity. The correct plasmid was then transformed into *Synechocystis* using triparental mating [44], resulting in the strain RE*phaAB*. The RE*phaAB* plasmid was also transformed in the strain Δ*pirC*, resulting in the strain PPT1 (Δ*pirC*-RE*phaAB*)*.*

**Table 3:** List of the oligonucleotides used in this study

Primer name	Sequence
psbA2fw2	gcttccagatgtatgctcttctgctcctgcaggtcgactcatttttccccattgccccaaaatac
psbA2rv2	gatacgatgacaacgtcagtcattttggttataattccttatgtatttg
RePhaABA2fw	caaatacataaggaattataaccaaaatgactgacgttgtcatcgtatc
RePhaABA2rv	atgaatgttccgttgcgctgcccggattacagatcctctatcagcccatgtgcaggccgccgttg

### Gas exchange limitation

When gas exchange limitation was applied, 10 ml of culture were transferred to a 15 ml reaction tube. The tube was closed and additionally sealed with several layers of parafilm. During the incubation, the reaction tubes were constantly shaken.

### Microscopy and staining procedures

To analyze the intracellular PHB granules, 100 µl of *Synechocystis* culture were centrifuged (10,000*g*, 2 min) and 80 µl of the supernatant discarded. Nile red (10 µl) was added and the pellet resuspended. From this mixture, 10 µl were dropped on an agarose-coated microscopy slide. For the detection, a Leica DM5500 B with an 100×/1.3 oil objective was used. An excitation filter BP 535/50 was used to detect Nile red stained granules.

### PHB quantification

To determine the intracellular PHB content, ~ 10 ml of cells were harvested by centrifugation (10 min at 4000*g*). The supernatant was discarded, and the remaining cell-pellet dried in a Speed-Vac for at least 2 h at 60 °C. The weight of the dried pellet was measured to determine the CDW. Next, 1 ml of concentrated sulfuric acid (18 M H_2_SO_4_) was added and the sample was boiled for 1 h at 100 °C. This process converts PHB to crotonic acid at a ratio of 1 to 0.893. The samples were diluted by transferring 100 µl to 900 µl of 14 mM H_2_SO_4_. Subsequently, the tubes were centrifuged for 10 min at 10,000*g*. Next, 500 µl of the supernatant were transferred to a new tube and 500 µl of 14 mM H_2_SO_4_ were added. The samples were centrifuged again and 400 µl of the clear supernatant was transferred into a glass vile for HPLC analysis. For this, a 100 C 18 column (125 by 3 mm) was used with 20 mM phosphate buffer at pH 2.5 for the liquid phase. As a standard, a dilution series of commercially available crotonic acid was used. The final amount of crotonic acid was detected at 250 nm.

### Electron microscopy

For electron microscopic pictures, *Synechocystis* cells were fixed and post-fixed with glutaraldehyde and potassium permanganate, respectively. Subsequently, ultrathin sections were stained with lead citrate and uranyl acetate [10]. The samples were then examined using a Philips Tecnai 10 electron microscope at 80 kHz.

### Purification of PHB

For the analysis of PHB, PPT1 cells were cultivated for four weeks in BG_11_ medium (without phosphorus and nitrogen) at light/dark regime. The cells were harvested by centrifugation for 10 min at 4000*g*. The cell pellet was resuspended in 15 ml freshly bought sodium hypochlorite solution (6%) and shaken over night at room temperature. The next day, the cell debris were centrifuged and washed with water (10 times), until the chlorine smell disappeared. Subsequently, the pellet was washed once with 80% ethanol and once with acetone.

### NMR and GPC

To characterize the chemical properties of PHB derived from PPT1, NMR spectra were recorded on a Bruker AVIII-400 spectrometer at ambient temperatures. As a control, an industrial standard PHB was used (BASF, Ludwigshafen, Germany). ^1^H and ^13^C NMR spectroscopic chemical shifts δ were referenced to internal residual solvent resonances and are reported as parts per million relative to tetramethylsilane. The tacticity of the polymer was analysed by ^13^C NMR spectroscopy according to literature [4]. As NMR solvent, CDCl_3_ was used (Sigma-Aldrich, Taufkirchen, Germany).

Measurements of polymer weight-average molecular weight (*M*_w_), number-average molecular weight (*M*_n_) and molecular weight distributions or dispersity indices (*Đ* = *M*_w_/*M*_n_) were performed via gel permeation chromatography (GPC) relative to polystyrene standards on an PL-SEC 50 Plus instrument from Polymer Laboratories using a refractive index detector. The analysis was performed at ambient temperatures using chloroform as the eluent at a flow rate of 1.0 mL min^−1^.

## Supplementary Information

**Additional file 1: Figure S1.** Drop plate assay of the WT and PPT1. Vegetative cells at an OD_750_ of 1 were diluted tenfold for five times (10^0^ to 10^4^, respectively). The dilutions were then dropped on a BG_11_ agar plate and grown under continuous light or light/dark rhythm for 7 or 12 days, respectively. The plate shown in the figure is representative of 3 individually grown biological replicates.

**Additional file 2: Figure S2.** PHB accumulation during vegetative growth. WT and PPT1 cells were sampled during exponential or stationary phase (OD ~ 1 and ~ 3, respectively) under continuous lighting. n.d. = not detectable. Each point represents a mean of three independent biological replicates.

**Additional file 3: Figure S3.** PHB production of WT (green) and PPT1 (blue) cells grown under continuous lighting. Cells shifted to nitrogen/phosphorus free medium (A) and with additional 100 mM NaHCO_3_ (B) or 10 mM acetate (C). Each point represents a mean of three independent biological replicates.

**Additional file 4: Figure S4.** PHB content of WT (green) and PPT1 (blue) cells grown in nitrogen/phosphorus free medium under light/dark regime. Dashed lines indicate growth in sealed vessels. Each point represents a mean of three independent biological replicates.

**Additional file 5: Figure S5.** ^1^H NMR (CDCl_3_, 400 MHz) spectrum of PHB derived from PPT1 compared to an industrial standard sample.

**Additional file 6: Figure S6.** ^13^C NMR spectrum (CDCl_3_, 101 MHz) of PHB derived from PPT1 compared to an industrial standard sample.

**Additional file 7: Figure S7.** ^13^C NMR spectrum to analyse the tacticity of PHB derived from PPT1. For comparison, industrial standard PHB (isotactic) and atactic PHB (produced from ß-butyrolactone via ring-opening polymerization) are shown.

## Data Availability

Not applicable.
