# Supplementary figures and images for: Maximizing PHB content in Synechocystis sp. PCC 6803: a new metabolic engineering strategy based on the regulator PirC
Source: Microb Cell Fact. 2020 Dec 22;19:231. doi: 10.1186/s12934-020-01491-1 (PMC7756911; doi:10.1186/s12934-020-01491-1)

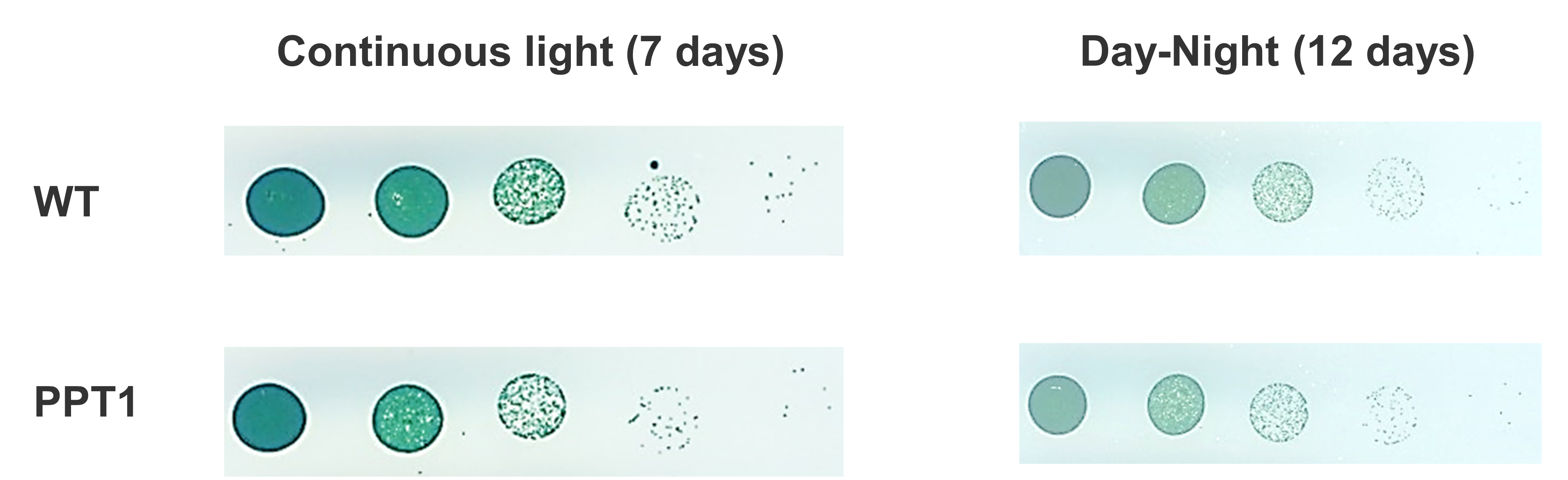

Supplement: Supplementary file 1 — Additional file 1: Figure S1. Drop plate assay of the WT and PPT1. Vegetative cells at an OD750 of 1 were diluted tenfold for five times (100 to 104, respectively). The dilutions were then dropped on a BG11 agar plate and grown under continuous light or light/dark rhythm for 7 or 12 days, respectively. The plate shown in the figure is representative of 3 individually grown biological replicates. [file 12934_2020_1491_MOESM1_ESM.tif]

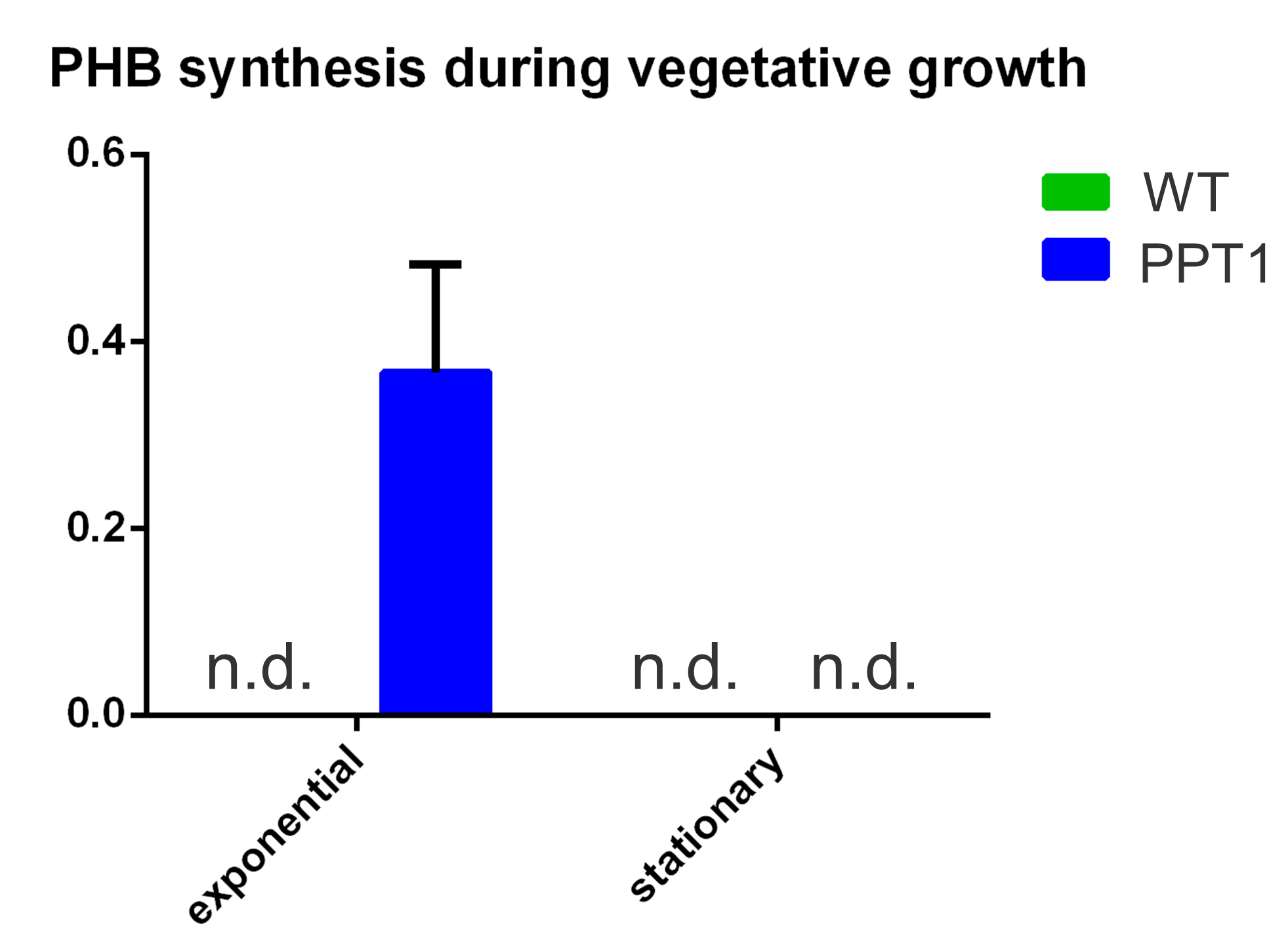

Supplement: Supplementary file 2 — Additional file 2: Figure S2. PHB accumulation during vegetative growth. WT and PPT1 cells were sampled during exponential or stationary phase (OD ~ 1 and ~ 3, respectively) under continuous lighting. n.d. = not detectable. Each point represents a mean of three independent biological replicates. [file 12934_2020_1491_MOESM2_ESM.tif]

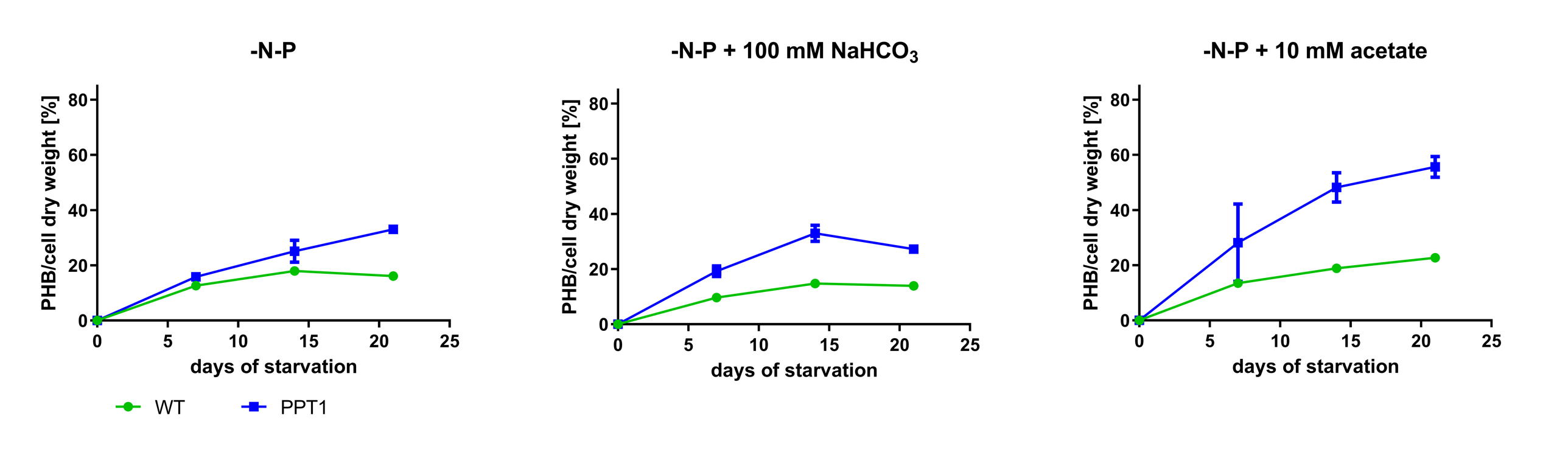

Supplement: Supplementary file 3 — Additional file 3: Figure S3. PHB production of WT (green) and PPT1 (blue) cells grown under continuous lighting. Cells shifted to nitrogen/phosphorus free medium (A) and with additional 100 mM NaHCO3 (B) or 10 mM acetate (C). Each point represents a mean of three independent biological replicates. [file 12934_2020_1491_MOESM3_ESM.tif]

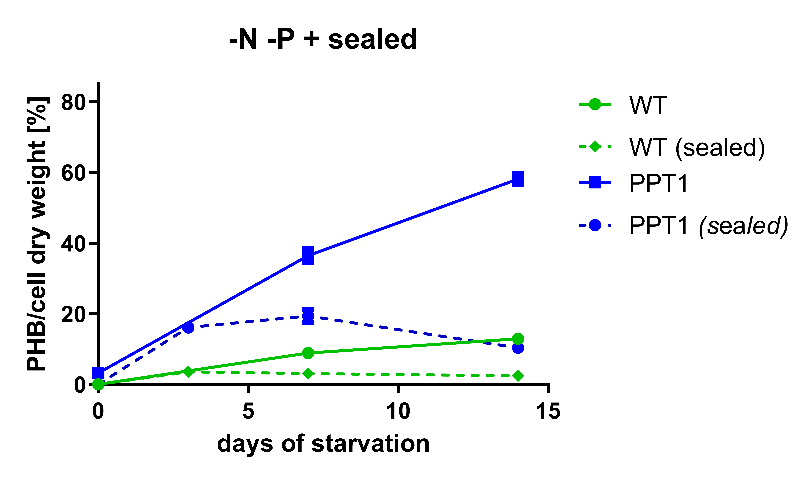

Supplement: Supplementary file 4 — Additional file 4: Figure S4. PHB content of WT (green) and PPT1 (blue) cells grown in nitrogen/phosphorus free medium under light/dark regime. Dashed lines indicate growth in sealed vessels. Each point represents a mean of three independent biological replicates. [file 12934_2020_1491_MOESM4_ESM.tif]

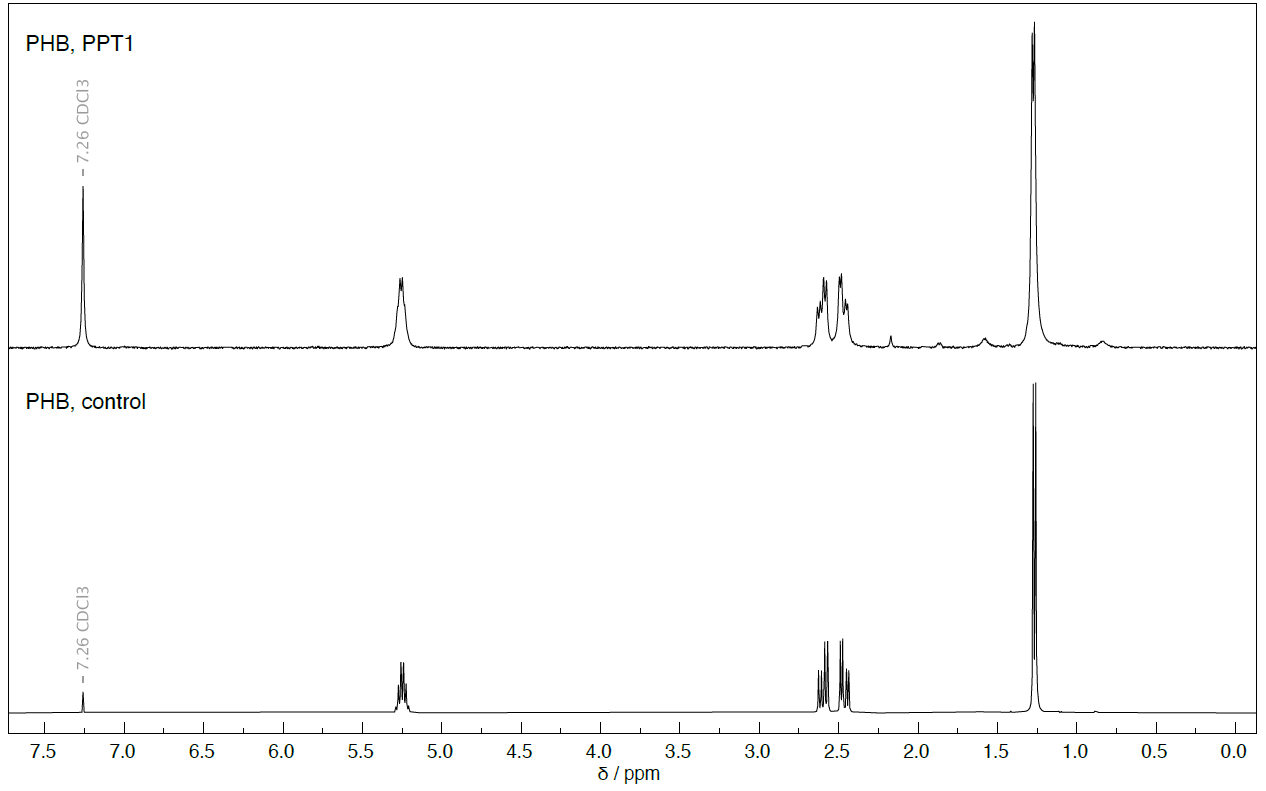

Supplement: Supplementary file 5 — Additional file 5: Figure S5. 1H NMR (CDCl3, 400 MHz) spectrum of PHB derived from PPT1 compared to an industrial standard sample. [file 12934_2020_1491_MOESM5_ESM.png]

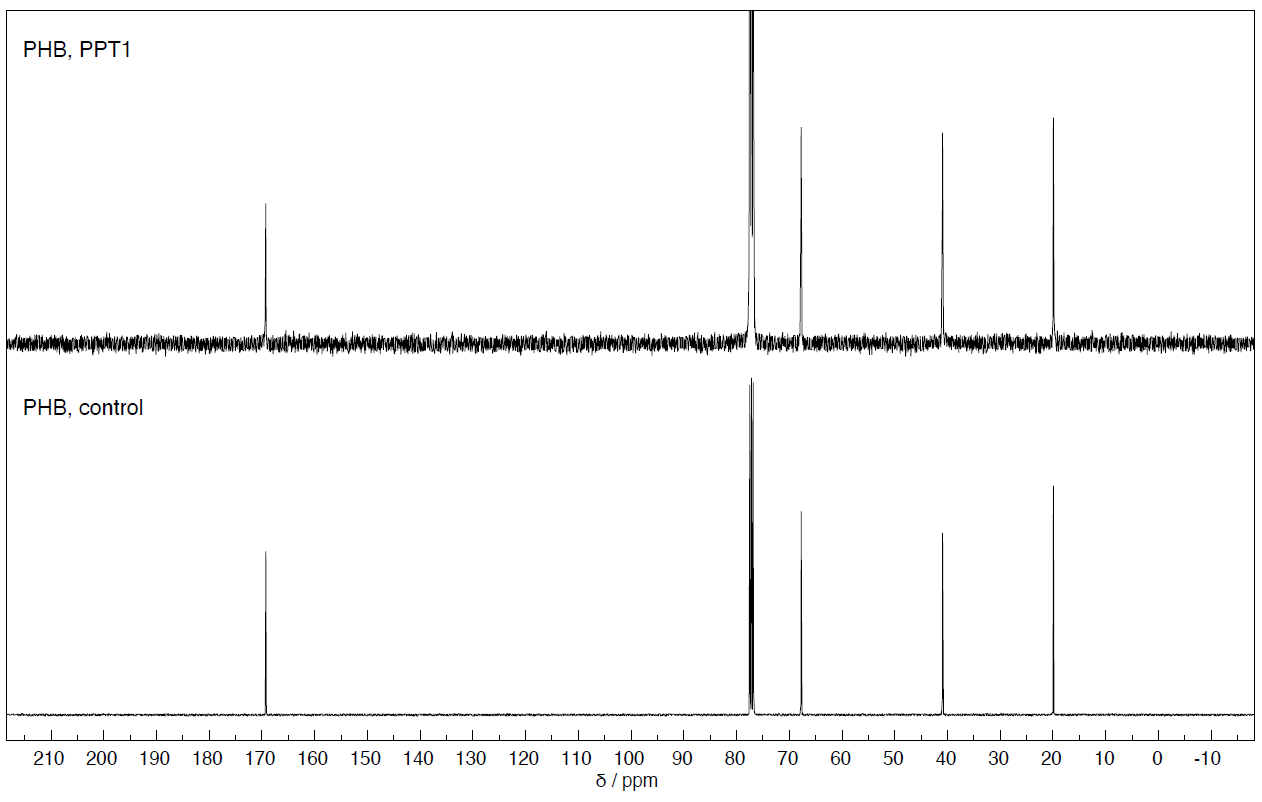

Supplement: Supplementary file 6 — Additional file 6: Figure S6. 13C NMR spectrum (CDCl3, 101 MHz) of PHB derived from PPT1 compared to an industrial standard sample. [file 12934_2020_1491_MOESM6_ESM.png]

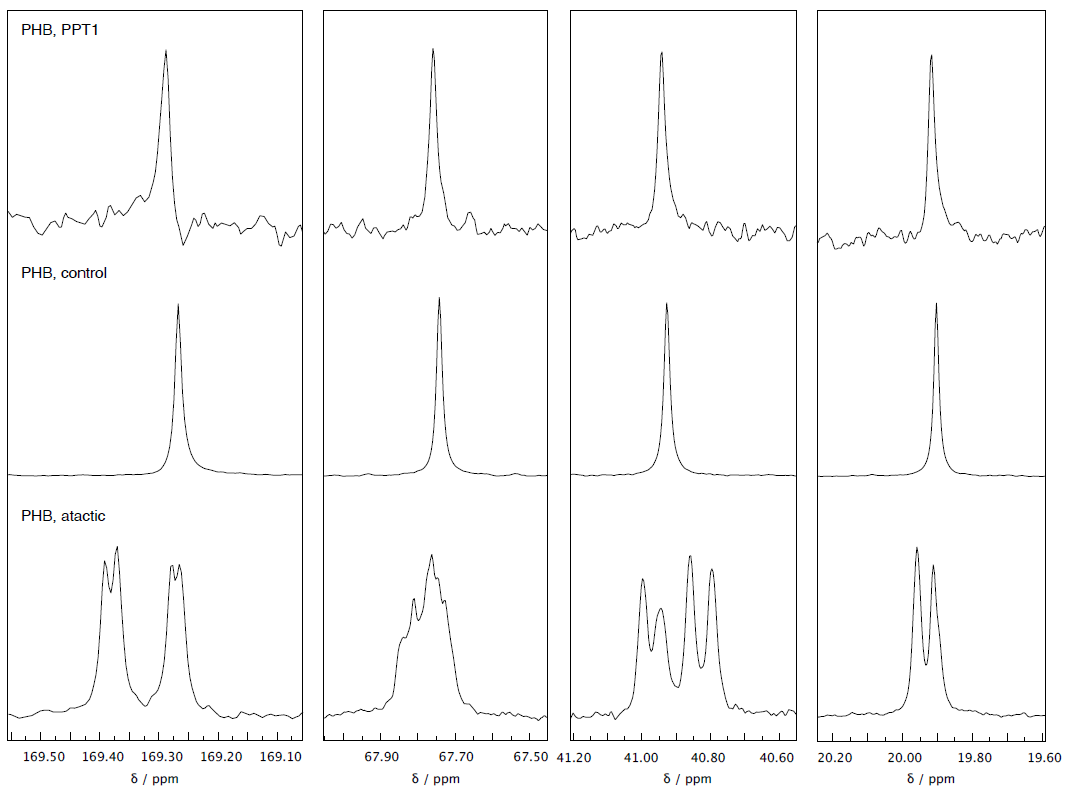

Supplement: Supplementary file 7 — Additional file 7: Figure S7. 13C NMR spectrum to analyse the tacticity of PHB derived from PPT1. For comparison, industrial standard PHB (isotactic) and atactic PHB (produced from ß-butyrolactone via ring-opening polymerization) are shown. [file 12934_2020_1491_MOESM7_ESM.png]
